# Clinical Analysis of Risk Factors for Mortality in Type A Acute Aortic Dissection: A Single Study From China

**DOI:** 10.3389/fcvm.2021.728568

**Published:** 2021-11-04

**Authors:** Hongliang Yuan, Zhenxing Sun, Yongxing Zhang, Wenqian Wu, Manwei Liu, Yali Yang, Jing Wang, Qing Lv, Li Zhang, Yuman Li, Mingxing Xie

**Affiliations:** ^1^Department of Ultrasound Medicine, Union Hospital, Tongji Medical College, Huazhong University of Science and Technology, Wuhan, China; ^2^Hubei Province Key Laboratory of Molecular Imaging, Wuhan, China

**Keywords:** acute type A aortic dissection, surgery, mortality, risk factors, retrospective

## Abstract

**Objective:** Acute type A aortic dissection (ATAAD) is a fatal condition that requires emergency surgery. The aim of the present study was to determine pre- and intra-operative risk factors for in-hospital mortality in patients with ATAAD.

**Methods:** Consecutive 313 patients with ATAAD who underwent emergency surgery at our hospital from February 2012 to February 2017 were enrolled in our study. Univariate and multivariate logistic regression analysis were performed to identify the pre-operative and intra-operative risk factors for in-hospital mortality.

**Results:** Of the 313 patients, 32 patients (10.2%) died. Compared with survivors, non-survivors had higher heart rate, serum potassium level and EuroSCORE II, and higher incidence of moderate to severe pericardial effusion, supra-aortic vessels involvement, myocardial ischemia and lower-extremity ischemia. As for surgery-related factors, the duration of surgery and cardiopulmonary bypass time were longer in non-survivors than survivors. In addition, non-survivors were more likely to undergo coronary-artery bypass graft compared with survivors. On multivariate analysis, elevated plasma potassium level (OR: 43.0, 95% CI: 3.8–51.5, *p* < 0.001), high incidence of supra-aortic vessels involvement (OR: 4.4, 95% CI: 1.5–7.0, *p* = 0.008) and lower-extremity ischemia (OR: 4.9, 95% CI: 1.6–6.9; *p* = 0.009), and longer duration of surgery (OR 6.0, 95% CI: 1.8–18.7, *p* = 0.000) and cardiopulmonary bypass time (OR: 3.7, 95% CI: 1.3–9.3, *p* = 0.001) were independently predictive of higher mortality in patients with ATAAD.

**Conclusions:** Supra-aortic vessels involvement, lower-extremity ischemia and elevated plasma potassium level are independent predictors of mortality in patients with ATAAD. A significant decrease in duration of surgery and cardiopulmonary bypass time is helpful to improve survival of patients.

## Introduction

Acute type A aortic dissection (ATAAD) is a life-threatening cardiovascular condition that requires emergent surgery. In recent years, the incidence of ATAAD has been increasing significantly due to high prevalence and poor control of hypertension (1). The development of advanced imaging technology had resulted in improving the diagnosis of ATAAD. Despite the improvement in medical management and surgical technique, the hospital mortality in patients with ATAAD remains high. Previous studies reported that the mortality was 16.9–18.4% after surgical repair of ATAAD (1–3). Therefore, it is necessary to recognize pre- and intra-operative risk factors for hospital mortality in patients with ATAAD.

Therefore, the aim of our study was to identify pre- and intra-operative risk factors for in-hospital mortality in patients with ATAAD.

## Methods

### Patients

Consecutive 321 patients with ATAAD who were admitted to the Union Hospital, Tongji Medical College, Huazhong University of Science and Technology from February 2012 to February 2017 were enrolled. Patients treated with endovascular repair (*n* = 6) or conservative treatment (*n* = 2) were excluded. Finally, 313 patients who underwent emergency surgery were included in our study. All patients were diagnosed by computed tomography angiography, magnetic resonance imaging or echocardiography. The study was approved by the institutional ethics board of Union Hospital Tongji Medical College, Huazhong University of Science and Technology.

### Clinical Data

Patients' demographic characteristics, medical histories, comorbidities, echocardiographic data, laboratory results, vessel involvement, organ ischemia, primary tear location, surgical type, duration of surgery, and outcomes were retrieved from electronic medical records. Medical histories included hypertension, diabetes, dyslipidemia, coronary artery disease and previous heart surgery. Echocardiographic data were composed of diameter of ascending aorta, moderate to severe pericardial effusion, aortic regurgitation and left ventricular ejection fraction. The diameter of ascending aorta was measured from the parasternal long-axis view. Left ventricular ejection fraction was assessed by M-mode echocardiography. Involvement of vessel branches included ascending aorta, aortic arch and supra-aortic vessels involvements. Surgical type encompassed ascending aorta replacement, aortic valve replacement, aortic arch replacement, elephant truck procedure, Coronary Artery Bypass Graft (CABG), and aortic sinus repair. Outcomes was defined as in-hospital mortality after operation. EuroSCORE II were calculated based on the prior method (http://www.euroscore.org/calc.html).

### Surgical Procedure

Operative techniques were composed of cardiopulmonary bypass (CPB), moderate hypothermia, circulatory arrest, and unilateral antegrade cerebral perfusion (u-ACP). The central venous pressure, bilateral radial artery pressure, electrocardiography, nasopharyngeal and rectal temperature and intermittent arterial blood gas analysis were monitored. Near-infrared spectroscopy (NIRS) was used to monitor cerebral saturation. When u-ACP was used, cerebral perfusion was performed through the right axillary artery. If the vessel was dissected, the true lumen of the branch vessel was cannulated. The flow rate for u-ACP was 10–15 mL/kg/min with perfusion pressure of 50–70 mmHg. After CPB was established, cooling was initiated. After clamping of the ascending aorta, cardiac arrest was accomplished with cold cardioplegic solution. Subsequently, the aortic root procedure depending on the severity and extent of the disease, including aortic root formation or Bentall procedure with or without CABG were performed.

### Statistical Analysis

Categorical variables are presented as frequencies and percentages. Normally distributed continuous variables are presented as the means and standard deviations. Non-normally distributed continuous variables are presented as medians with quartiles. Continuous variables were compared using the *t*-test or Mann-Whitney U test. Categorical data were compared using the Fisher's exact or Chi-square tests. Receiver-operating characteristic (ROC) curves were used to obtain diagnostic cutoff values, as well as specificity and sensitivity. Estimations of the risk factors of mortality were performed using univariate and multivariate logistic regression models. To avoid the interaction between the pre-operative and intra-operative variable when the pre-operative and intra-operative variables were enrolled together in multivariate logistic regression analysis, we analyzed the pre- or intra-operative data independently. The possible pre-operative risk factors, including demographics, comorbidities, echocardiographic measurements, vessel involvement and laboratory results, were included in the pre-operative univariate logistic regression analysis. The potentially intra-operative predictors of higher mortality, including surgical type and duration of surgery, were entered into in the intra-operative univariate logistic regression analysis. Variables with *p* values <0.05 in univariate logistic regression analysis were entered into multivariate logistic regression models. The results of the logistic regressions are presented as odds ratio (OR) with confidence intervals (CI). The statistical analyses were performed with SPSS version 21.0 (SPSS Inc., Chicago, IL, USA). A 2-sided *p* value <0.05 was considered to indicate statistical significance.

## Results

A total of 313 patients were included in our study. Pre-operative characteristics of patients with ATAAD are summarized in Table 1. The mean age of patients with ATAAD was 48 years, and 264 (84%) were men. Only unilateral antegrade cerebral perfusion was performed in every patient with supra-aortic vessels involvement. NIRS was used in the intra-operative assessment of brain perfusion. Of these patients, 32 patients (10.2%) died. Non-survivors had higher heart rate and plasma potassium level than survivors. Compared with survivors, non-survivors displayed higher prevalence of moderate to severe pericardial effusion, involvement of supra-aortic vessels, myocardial ischemia and lower limb ischemia. There were no significant differences in age, sex, systemic arterial pressure, comorbidities (hypertension, diabetes, dyslipidemia and coronary artery disease), antihypertensive drug use between non-survivors and survivors. With regard to echocardiographic data, the diameter of ascending aorta, left ventricular ejection fraction and the prevalence of aortic regurgitation were similar in survivors and non-survivors. In addition, leukocyte, neutrophil and platelets counts, and creatinine level did not differ between survivors and non-survivors.

**Table 1:** Pre-operative characteristics of patients with ATAAD.

	**Survivors**	**Non-survivors**	***P*-value**
	**(*n* = 281)**	**(*n* = 32)**	
Age (years), mean ± SD	48 ± 10	48 ± 11	0.87
Sex			
Female, *n* (%)	47 (15.0)	2 (0.6)	0.12
Male, *n* (%)	234 (85.0)	30 (99.4)	
Medical histories, n (%)			
Hypertension	168 (59.8)	20 (62.5)	0.77
Diabetes	4 (1.4)	2 (6.3)	0.23
Dyslipidemia	10 (3.6)	3 (9.4)	0.27
Coronary artery disease	30 (10.7)	6 (18.8)	0.29
Smoking history	137 (48.8)	19 (59.4)	0.26
Alcohol history	113 (40.2)	14 (43.8)	0.70
Previous heart surgery	3 (0.7)	2 (6.3)	0.08
Antihypertensive drugs			
ACEI	43 (0.36)	6 (0.4)	0.73
CCB	39 (0.32)	5 (0.33)	1.0
β-blocker	27 (0.22)	3 (0.2)	1.0
ARB	12 (0.1)	1 (0.07)	1.0
Admission vital signs			
Heart rate (beats per minute)	80 ± 15	87 ± 16	0.01
Systolic blood pressure (mm Hg)	135 ± 29	133 ± 26	0.61
Diastolic blood pressure (mm Hg)	72 ± 19	73 ± 12	0.68
Echocardiographic measurements			
DAA (mm), mean ± SD	47 ± 8	47 ± 5	0.94
Moderate to severe PE	163 (58.0)	25 (78.1)	0.03
LVEF (%), mean ± SD	62.7 ± 5.4	60.2 ± 6.1	0.12
Aortic regurgitation	155 (55.2)	15 (46.9)	0.37
Laboratory results (maximum value pre-surgery), mean ± SD			
Leukocyte (10^9^)	12.0 ± 4.1	12.7 ± 4.9	0.36
Platelets (10^9^)	168.0 ± 66.3	165.3 ± 65.1	0.83
Creatinine (μmol/l)	106.4 ± 79.7	125.9 ± 59.5	0.19
Neutrophil (10^3^/μl)	13.5 ± 57.9	10.9 ± 4.7	0.80
Plasma potassium (mg/dl)	3.8 ± 0.4	4.7 ± 0.4	<0.001
Involvement of vessel branches			
Ascending aorta	280 (99.6)	32 (100.0)	0.91
Aortic arch	257 (91.5)	32 (100.0)	0.17
Supra-aortic vessels	160 (56.9)	26 (81.3)	<0.01
Organ ischemia			
Lower limb ischemia	16 (5.7)	11 (34.4)	<0.01
Myocardial ischemia	28 (10.0)	9 (28.1)	<0.01
Primary tear location, n (%)			
Ascending aorta	155 (55.2)	15 (46.9)	0.37
Aortic arch	71 (25.3)	5 (15.6)	0.23
Descending aorta	24 (8.5)	9 (28.1)	0.32
Unknown	31 (11.0)	3 (9.4)	0.78
EuroSCORE II, (%)	4.6 ± 1.5	12.1 ± 3.9	<0.001

*DAA, diameter of ascending aorta; PE, pericardial effusion; ACEI, angiotensin converting enzyme inhibitors; CCB, calcium channel blockers; ARB, angiotensin receptor blocker; LVEF, left ventricular ejection fraction*.

Intra-operative characteristics of patients with ATAAD are presented in Table 2. Compared with survivors, non-survivors had longer operation time and cardiopulmonary bypass time (CPBT). Non-survivors were more likely to undergo CABG than survivors. There was no statistically significant difference in aorta occlusion time, circulatory arrest time, lowest rectal temperature and cerebral perfusion time between non-survivors and survivors. Additionally, non-survivors had a similar treatment with ascending aorta replacement, aortic valve replacement, half or whole arch replacement, elephant trunk technique and aortic sinus repair as survivors.

**Table 2:** Procedure characteristics of patients with ATAAD.

	**Survivors**	**Non-survivors**	***P*-value**
	**(*n* = 281)**	**(*n* = 32)**	
Procedure type, n (%)			
Ascending aorta replacement	147 (52.3)	21 (65.6)	0.15
Aortic valve replacement	135 (48.0)	11 (34.4)	0.14
Aortic arch replacement	263 (93.6)	32 (100.0)	0.28
Elephant truck procedure	247 (87.9)	32 (100.0)	0.07
CABG	30 (10.7)	9 (28.1)	0.01
Aortic sinus repair	52 (18.5)	8 (25.0)	0.38
Duration of procedure, mean ± SD			
Procedure time (h)	8.3 ± 2.1	9.8 ± 1.9	<0.001
CPB time (min)	230 ± 45	293 ± 92	0.01
Cross-clamp time (min)	125 ± 26	128 ± 26	0.66
HCA time (min)	22 ± 7	26 ± 10	0.10
Lowest rectal temperature (°C)	23.7 ± 1.5	24.1 ± 1.3	0.10
Cerebral perfusion time (min)	22 ± 6	24 ± 8	0.55

*CABG, coronary artery bypass graft; CPB, cardiopulmonary bypass; HCA, hypothermic circulatory arrest*.

**Figure 1:**
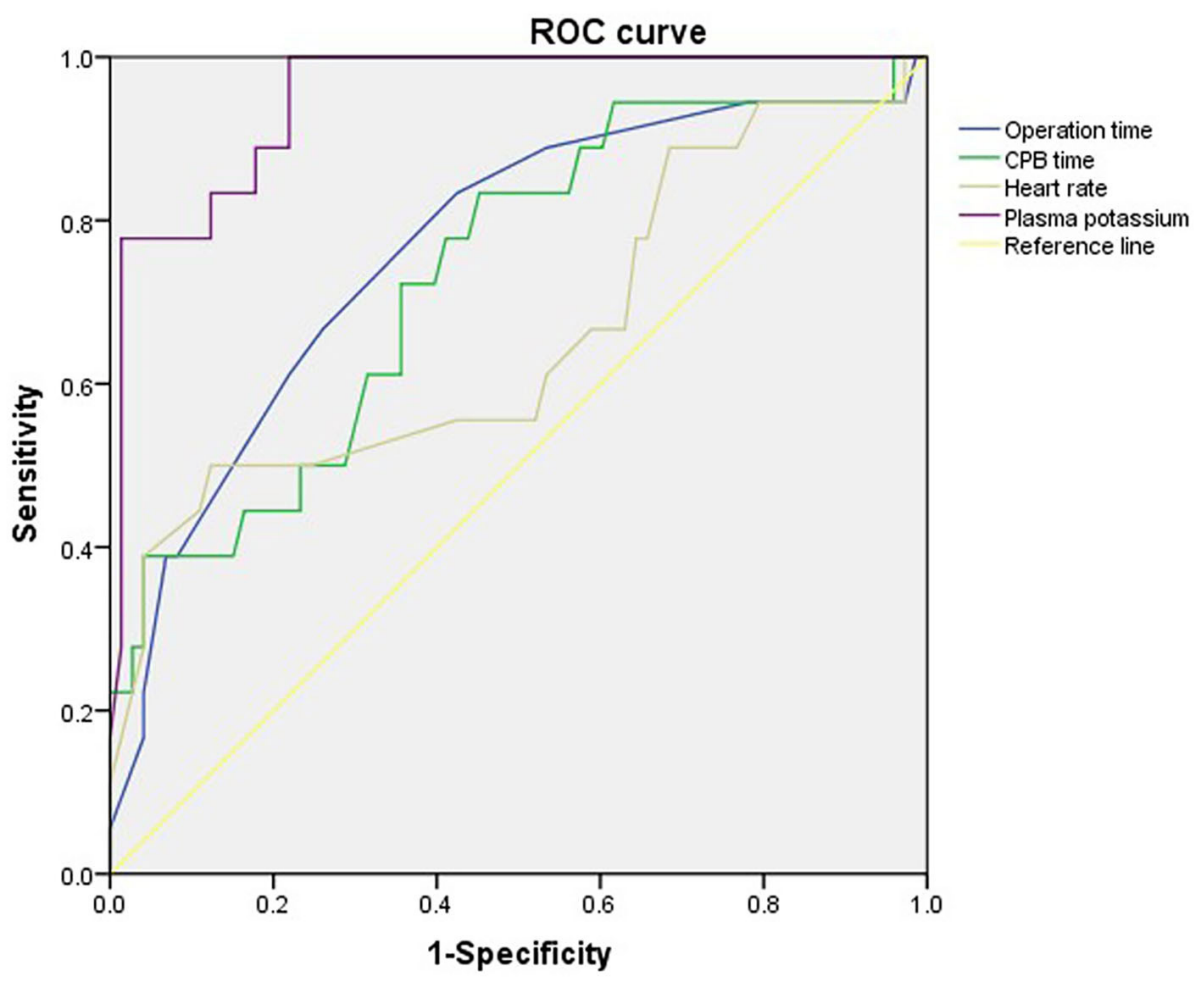
Receiver-operating characteristic curves of operation time, extracorporeal circulation time, admission heart rate and blood potassium level to identify mortality in ATAAD patients.

ROC curves were used to determine the optimal cut-off values for operation time, extracorporeal circulation time, admission heart rate and blood potassium level to identify mortality in ATAAD patients (Table 3; Figure 1). ROC analysis revealed that duration of operation > 9.5 h [area under the curve (AUC): 0.73], CPBT > 227 min (AUC: 0.72), heart rate > 82 beats/min (AUC: 0.65) and plasma potassium > 4.4 mmol/L (AUC: 0.92) were associated with in-hospital mortality in patients with ATAAD.

**Table 3:** ROC curves to obtain diagnostic cut-off values.

**Variables**	**Cut-off value**	**Sensitivity (%)**	**Specificity (%)**	**AUC**
Duration of operation (h)	9.5	58.1	78.4	0.73
CPB time (min)	227	83.3	52.7	0.72
Heart rate (beats/min)	82	53.1	72.6	0.65
Plasma potassium (mmol/l)	4.4	72.0	97.2	0.92

*CPB, cardiopulmonary bypass*.

Univariate logistic regression analysis including pre-operative variables revealed that plasma potassium > 4.4 mmol/L, supra-aortic branch involvement, lower limb ischemia, and myocardial ischemia were predictors of higher mortality in patients with ATAAD. Multivariable logistic regression analysis demonstrated that plasma potassium > 4.4 mmol/L (OR: 43.0, 95% CI: 3.8–51.5, *p* < 0.001), supra-aortic branch involvement (OR: 4.4; 95% CI: 1.5–7.0, *p* = 0.008), and lower limb ischemia (OR: 4.9, 95% CI: 1.6–6.9, *p* = 0.009) were independently predictive of in-hospital mortality (Table 4).

**Table 4:** Logistic analysis of pre-operative risk factors for mortality in ATAAD.

	**Univariate**			**Multivariate**		
**Variables**	**OR**	**95% CI**	***P*-value**	**OR**	**95% CI**	***P*-value**
Age (years)	1.0	0.003–0.03	0.87			
Sex	3.0	1.1–2.2	0.14			
Heart rate > 82 (beats/min)	1.9	0.7–3.2	0.07			
Systolic blood pressure (mmHg)	1.0	−0.03–0.27	0.61			
Diastolic blood pressure (mmHg)	1.0	0.003–0.08	0.78			
Hypertension	3.0	1.1–3.9	0.05			
Smoking history	1.5	0.4–1.1	0.29			
Dyslipidemia	2.8	1.0–2.3	0.13			
Diabetes	4.6	1.5–3.0	0.09			
Previous heart surgery	6.2	1.8–3.8	0.051			
Plasma potassium > 4.4 (mmol/l)	45.3	3.8–64.6	<0.001	43.0	3.8–51.5	<0.001
Creatinine (μmol/l)	1.0	0.002–1.6	0.20			
Neutrophil (10^3^/l)	1.0	−0.002–0.06	0.81			
Platelets (10^9^)	1.0	−0.001–0.05	0.83			
Moderate to severe pericardial effusion	2.4	0.9–3.8	0.051			
LVEF (%)	0.9	−0.08–1.8	0.18			
Aortic regurgitation	0.9	−0.06–0.03	0.87			
DAA (mm)	1.0	−0.002–0.01	0.94			
Supra-aortic vessels involvement	5.5	1.7–13.2	<0.001	4.4	1.5–7.0	0.008
Myocardial ischemia	3.0	1.1–5.9	0.02	2.2	0.8–1.5	0.22
Lower-extremity ischemia	5.0	1.6–15.2	<0.001	4.9	1.6–6.9	0.009

*DAA, diameter of ascending aorta; LVEF, left ventricular ejection fraction*.

Univariate logistic regression analysis including intra-operative variables showed that elephant truck procedure, CABG, operation time > 9.7 h, CPBT > 227 min could predict higher mortality in patients with ATAAD. Multivariable logistic regression analysis revealed that operation time >9.7 h (OR: 6.0, 95% CI: 1.8–18.7, *p* < 0.001), CPBT > 227 min (OR: 3.7, 95% CI: 1.3–9.3, *p* = 0.002), and elephant truck procedure (OR: 4.1, 95% CI: 1.4–6.9, *p* = 0.008) were independent predictors of in-hospital mortality (Table 5).

**Table 5:** Logistic analysis of intra-operative risk factors for mortality in ATAAD.

	**Univariate**			**Multivariate**		
**Variables**	**OR**	**95% CI**	**P-value**	**OR**	**95%CI**	**P-value**
Ascending aorta replacement	1.8	0.6–2.3	0.13			
Elephant truck procedure	3.4	1.2–5.8	0.02	4.1	1.4–6.9	0.008
Aortic valve replacement	0.6	−0.57–2.1	0.15			
Aortic arch replacement	2.2	0.8–3.7	0.14			
CABG	3.3	1.2–7.3	0.007	1.9	0.6–1.5	0.23
Operation time > 9.7 (h)	5.3	1.7–18.4	<0.001	6.0	1.8–18.7	<0.001
CPB time > 227 (min)	4.4	1.5–13.0	<0.001	3.7	1.3–9.3	0.002
HCA time (min)	1.0	0.03–0.6	0.45			
Cross-clamp time (min)	1.0	0.004–0.2	0.65			
Lowest rectal temperature (°C)	1.5	0.4–3.8	0.05			
Cerebral perfusion time (min)	1.0	0.03–0.4	0.54			

*CPB, cardiopulmonary bypass; HCA, hypothermic circulatory arrest; CABG, coronary artery bypass graft*.

## Discussion

In this study of 313 consecutive patients with ATAAD who underwent emergency surgery from February 2012 and February 2017, the overall hospital mortality was 10.2%. Although the hospital mortality was lower than that of previous studies (1–3), it is still unacceptable. It is necessary to summarize the risk factors for in-hospital mortality in patients with ATAAD. Our results indicated that pre- and intra-operative risk factors for in-hospital mortality in patients with ATAAD were elevated level of plasma potassium, higher incidence of supra-aortic vessels involvement and lower-extremity ischemia, and longer duration of operation and cardiopulmonary bypass time.

The present study showed that supra-aortic vessels involvement was a risk factor for hospital death. The extension of ATAAD with involvement of the supra-aortic branches largely determined the pre-operative state of the patients and influenced the post-operative outcome. Mortality may increase rapidly if the supra-aortic vessels are involved in the dissection process. Patients with ATAAD with supra-aortic vessel involvement have a higher risk of post-operative stroke. Understandably, anastomosis of branch vessels is required during surgery in these patients, which prolonged the time of extracorporeal circulation and operation, and further may lead to diminished blood supply to the brain. Thus, cerebral malperfusion further increase in-hospital mortality (1, 4). In the current study, we found that 186 patients had supra-aortic vessels involvement. Twenty six of these patients (14.0%) died during hospitalization. Thereby, an appropriate protection procedure is important to prevent irreversible brain injury. The various cerebral protection (antegrade/ retrograde; monolateral/bilateral) can be used during aortic arch surgery. The unilateral antegrade cerebral perfusion was performed during aortic arch surgery in this study. The relative benefits of unilateral antegrade cerebral perfusion compared with bilateral antegrade cerebral perfusion as cerebral perfusion strategies remained undetermined. Tong et al. revealed that bilateral antegrade cerebral perfusion did not significantly reduce 30-days mortality and permanent neurologic dysfunction compared to unilateral antegrade cerebral perfusion (5). However, Misfeld et al. showed that early mortality and medium-term survival was not affected by the type of cerebral protection used (6). In addition, Zierer et al. demonstrated that unilateral antegrade cerebral perfusion had the equal brain protection compared to bilateral antegrade cerebral perfusion (7). More and more data show that unilateral cerebral perfusion in moderate hypothermia is safe when performed during NIRS monitoring and that avoiding deep hypothermia preserves cerebral autoregulation blood flow, resulting in an optimal unilateral perfusion. Only unilateral antegrade cerebral perfusion was performed in every patient with supra-aortic vessels involvement during February 2012 to February 2017 in this study. So, we did not determine the different impact of the type of cerebral protection on clinical outcomes in cases of an aortic arch surgery and involvement of supra-aortic vessels in our hospital. It deserves further investigation whether unilateral antegrade cerebral perfusion is superior to bilateral antegrade cerebral perfusion in patients with ATAAD with supra-aortic vessels involvement.

In this study, non-survivors had the higher incidence of lower limb ischemia than survivors. Moreover, multivariate logistic regression analysis showed that lower limb ischemia was a risk factor for mortality in patients with ATAAD, which was similar as previous studies (8, 9). Poor perfusion of the lower extremities can lead to serious post-operative complications (10). In our study, lower-extremity ischemia was diagnosed in 27 patients with no palpable pulses in the femoral artery before surgery. Eleven of these patients (40.7%) died during hospitalization. Uchida et al. showed that improvements of the blood supply of lower-extremity by draining the brachial arterial blood to the ischemic lower limb arteries, may significantly improve symptoms (11). Whether this strategy should be mandatory is highly controversial. Preece et al. demonstrated that inferior-limb ischemic artery reperfusion before aortic repair increased intra-operative mortality in ATAAD patients (12).

In addition to supra-aortic vessels involvement and lower limb ischemia, elevated level of plasma potassium was found to be another independent risk factor of higher in-hospital mortality in patients with ATAAD. Indeed, our study demonstrated that non-survivors had higher blood potassium level than survivors. Moreover, the current observation demonstrated that the optimal cut-off value of plasma potassium level for predicting higher mortality in patients with ATAAD was over 4.4 mmol/L. Patients who had higher level of plasma potassium above the cutoffs had increased risk of mortality. Potassium serum levels within the high normal range (>4.4 to < 5.0 mmol/L), which were frequently ignored by clinicians in the emergency department, were associated with higher in-hospital mortality. This finding is consistent with the results of Chen et al., who found that the blood potassium level other than 3.5 to 4.5 mmol/L at admission was related to higher in-hospital and long-term mortality in ATAAD patients (13). Previous studies found a U-shaped relationship between serum potassium levels at admission and in-hospital mortality, which potassium levels outside the interval of <3.5 to 4.5 mmol/L were associated with higher risk in all-cause mortality (13, 14). The risk of cardiovascular mortality increases with the elevated potassium levels, due to the fact that the heart is more susceptible to potassium fluctuation than other tissues. An increase in serum potassium level is associated with reduced ventricular excitability and has been shown to cause increased diastolic threshold of excitability in experimental animals and humans (15, 16), which would be associated with an increased prevalence of cardiac arrhythmias. A large body of evidences have demonstrated that even a mild change within the normal range is associated with higher mortality in patients with various cardiovascular diseases, such as hypertension (17), acute coronary syndrome (14, 18), and heart failure (19, 20), which reveal that the optimal blood potassium level may be different from the definite clinical normal range in diverse cardiovascular diseases. ATAAD patients with more severe circumstances (such as use of antihypertensive drugs, hemorrhage and hemolysis) may experience a serum potassium disturbance, which is similar to that in other cardiovascular diseases. However, current guidelines do not underscore potassium management in patients with aortic diseases (21, 22). Our results demonstrated that higher potassium levels at admission had an adverse effect on the in-hospital mortality of patients with ATAAD, and accordingly, more active management may be necessary.

The duration of extracorporeal circulation was significantly longer in non-survivors than survivors. Furthermore, a multivariable analysis showed that the duration of surgery and the duration of extracorporeal circulation were risk factors for in-hospital mortality. Our findings are consistent with previous studies (23). A long operation time reflects the severity of ATAAD; moreover, it had an adverse effect on organ perfusion. Therefore, these may be the reason why longer duration of operation is associated with unfavorable outcomes in patients with ATAAD.

Previous researches indicated that myocardial ischemia was a risk factor for in-hospital mortality in patients with ATAAD. In our study, univariate regression analysis demonstrated that myocardial ischemia was a predictor of mortality in patients with ATAAD. However, in multivariate logistic regression analysis, myocardial ischemia was no longer significantly predictive of in-hospital mortality. In our series, 37 patients had myocardial infarction before surgery, and 9 of them (24.3%) died during hospitalization. This may be the reason that most patients with myocardial ischemia were simultaneously treated with coronary artery bypass graft during the aortic replacement, which markedly improved myocardial ischemia and the prognosis.

In our study, 188 patients with ATAAD presented with medium to large amounts of pericardial effusion. Non-survivors had a higher incidence of medium to large amounts of pericardial effusion than survivors. However, univariate and multiple regression analysis revealed that moderate to severe pericardial effusion could not predict higher in-hospital death in patients with ATAAD. This finding was not in line with the study of Santi Trimarchi et al., which demonstrated that pericardial tamponade was an independent predictor of post-operative mortality in patients with ATAAD (8).

Our study revealed that elephant truck procedure was a risk factor of mortality in patients with ATAAD. This result was not in keeping with the observation of Goda et al., which indicated that various surgical methods did not affect the early post-operative mortality in ATAAD patients (9). However, Tan et al. found that simultaneous aortic valve replacement or Bentall surgery in ATAAD patients was a protective factor for post-operative mortality (23). Future studies are needed to confirm the effect of various surgical treatment on outcomes in patients with ATAAD.

### Limitations

Our study had some limitations. First, this was a retrospectively single-center study, extrapolation of our findings could be affected by local bias. In addition, sample size of this study is relatively limited. Future multicenter studies with larger sample sizes are needed to verify our findings. Another limitation of our study is that we did not determine the different impact of the type of cerebral protection on clinical outcomes in cases of an aortic arch surgery and involvement of supra-aortic vessels, because that only unilateral antegrade cerebral perfusion was performed in every patient with supra-aortic vessels involvement in our study. The effect of cerebral protection type on outcomes in patients with supra-aortic vessels involvement is an interesting topic. Future study that investigates the different impact of cerebral protection type on clinical outcomes could be the next step. Finally, the numbers of non-survivors were small, the exiguous number of the mortality group and the great numeric difference between the two groups could represent a statistical limitation for a solid conclusion based on this study.

## Conclusion

Supra-aortic vessels involvement, lower-extremity ischemia and elevated plasma potassium level are pre-operative risk factors for in-hospital mortality in patients with ATAAD. Among intra-operative factors, longer operation time and cardiopulmonary bypass time are associated with increased in-hospital mortality. For further improvement of outcomes, quicker diagnosis, more appropriate pre-operative management and minimizing any delay in surgery are mandatory.

## Data Availability Statement

The original contributions presented in the study are included in the article/supplementary material, further inquiries can be directed to the corresponding author/s.

## Ethics Statement

The studies involving human participants were reviewed and approved by the Ethical Committee of Tongji Medical College of Huazhong University of Science and Technology. Written informed consent was not required for this study, in accordance with the local legislation and institutional requirements.

## Author Contributions

MX and LZ contributed to the conception of the study. HY, ZS, and YL analyzed and interpreted the clinical data and imaging findings, and they were major contributors in writing the manuscript. YZ, WW, and ML helped perform the analysis with constructive discussions. All authors read and approved the final manuscript.

## Funding

This work was supported by National Natural Science Foundation of China (Grant Nos. 81727805, 81701716, 81922033), the Key Research and Development Program of Hubei (Grant No. 2020DCD015), the Fundamental Research Funds for the Central Universities (Grant No. 5003530082), and the Shenzhen Science and Technology under (Grant No. SGDX20190917094601717).

## Conflict of Interest

The authors declare that the research was conducted in the absence of any commercial or financial relationships that could be construed as a potential conflict of interest.

## Publisher's Note

All claims expressed in this article are solely those of the authors and do not necessarily represent those of their affiliated organizations, or those of the publisher, the editors and the reviewers. Any product that may be evaluated in this article, or claim that may be made by its manufacturer, is not guaranteed or endorsed by the publisher.
